# Training interprofessional teams in geriatric emergency medicine: A modified team-based learning approach

**DOI:** 10.1016/j.heliyon.2024.e25099

**Published:** 2024-02-07

**Authors:** Adeviyye Karaca, Mustafa Daloğlu, Deniz Kılıç, Ramazan Sivil, Mustafa Keşaplı, Mustafa Kemal Alimoğlu

**Affiliations:** a Department of Emergency Medicine, University of Health Science Antalya Training and Research Hospital, Antalya, Turkey; b Department of Medical Education, Akdeniz University, Antalya, Turkey

**Keywords:** Interprofessional learning, Team-based learning, Geriatric emergency medicine, Long-term retention, Learner satisfaction

## Abstract

**Background:**

Older adults deserve special healthcare provision in every branch of medicine. Turkey currently does not have geriatric emergency medicine (GEM) subspecialty training. Thus, interprofessional training for healthcare professionals involved in GEM services is required. Team-based learning (TBL) seems suitable to implement such training. We aimed to develop and implement a training program for healthcare professionals engaged with GEM services, and evaluate the program considering teacher and learner satisfaction and knowledge retention.

**Methods:**

This was a design-based study in which a one-day GEM training program was developed based on the literature and expert opinions. The program was applied to 54 physicians, 98 nurses, 70 health officers, and 102 paramedics using a modified version of TBL. Teams included at least one representative from each profession. TBL was modified by adding a 1-h lecture and eliminating peer evaluation. Feedback forms, individual and group tests of TBL, and a retention test conducted six months later were used for program evaluation.

**Results:**

The mean group test score was higher than that of individual tests in all professions. Physicians’ individual test scores were higher than those of other professions, but this difference disappeared in the group test. The retention test mean score was higher than the individual test mean score but lower than that of the group test. Teacher and learner satisfaction was high.

**Conclusion:**

We implemented a training program using a modified TBL approach to teach GEM to relevant healthcare professionals; it yielded promising results regarding knowledge gain and retention, as well as teacher and learner satisfaction. The instruction design and method used in this study can be applied to multidisciplinary team training.

## 1 Introduction

The older adult population (aged over 65 years) is increasing worldwide. According to the US Census Bureau, for the first time in history, the population aged 65 and over is expected to outnumber the population under 18 by 2034 [1]. In addition, between 2016 and 2060, the US population was expected to increase by approximately 25%; furthermore, the population aged 65 and over was expected to increase approximately two times, the population aged 85 and over three times, and the population aged 100 and over seven times [2]. Global population aging clearly reveals the need for special healthcare service provision for older adults, with different medical disciplines forming sub-branches specific to their health [3].

According to the Turkish Statistical Institute, the older adult population was approximately six million (8% of the total) in 2016, and reached 7.5 million (9.1% of the total) in 2021, demonstrating an increase by 21.9% in five years. Population projections suggest that the older adult population rate will be 12.9% in 2030, 16.3% in 2040, and 22.6% in 2060. According to the 2020–2021 data, overall life expectancy at birth was 78.3 years (75.6 years for men and 81 years for women) in Turkey [4].

One-third of the patients admitted to the emergency department are aged over 65, and this figure is projected to increase over the years [5]. The costs, as well as the hospitalization and mortality rates, associated with older adults in the emergency department are higher compared to young adults [6]. The mortality rate of patients aged 60 and over hospitalized in the emergency department is 21% [7]. Increased visits to the emergency department contribute to decreased quality of patient care, delays in treatment initiation, increased length of hospitalization, less adherence to accepted clinical guidelines, and increased overall costs [8].

The increase in the older adult population and their more frequent hospitalizations compared to the young pose challenges to healthcare systems as they often present with more than one chronic disease, are at risk of inappropriate medication and polypharmacy, and have complex social and physical difficulties. Older adults, who have unique disease presentations, needs, trends, and outcomes, come into contact with the healthcare system at many points, but perhaps none as frequently or as important as the emergency room [8].

The concept of geriatric emergency medicine (GEM) was first introduced in the US in 1996 as a sub-branch of emergency medicine. Subsequently, training and standardization have been developed. GEM models have been developed so that patients can access appropriate and high-quality emergency healthcare services in hospitals [[9], [10], [11]]. The Geriatric Emergency Service Guide was published by the American Emergency Medicine Association in 2014 and a relevant accreditation process was initiated [12]. After emergency medicine was accepted as a separate specialty in Europe in 2009, the GEM curriculum was published in 2016 under the leadership of the European Emergency Medicine Association's GEM branch [13].

Geriatric patient care requires appropriate triage, trained healthcare personnel, equipment designed according to special needs, and special planning and procedures/protocols. Thence, patient-specific evaluation, diagnosis, and treatments can be more permanent; patients can reap the maximum benefit from health services; and unnecessary health expenses can be prevented. Therefore, it would be beneficial for emergency services to develop “geriatric-friendly” programs [14].

Thereupon, geriatric emergency services started to be established in European countries. At the time this study was initiated, there was no geriatric emergency service model and practice in Turkey. The most concrete step taken in this regard is the ERASMUS + project, implemented with the aim of establishing the first geriatric emergency department to provide qualified and high-level emergency health services to older adults, and to develop a training program to train relevant staff from different professions.

The gold standard of care for older adults with fragility is comprehensive geriatric assessment [15]. Fragility in older adults manifests as simultaneous problems in more than one medical specialty [16]. This situation requires a multifaceted approach and, therefore, a team that can handle different medical approaches at the same time. Geriatric medicine is not a medical profession focused on a specific field. Thus, the main element of healthcare for older adults is the multidisciplinary and multiprofessional team that coordinates care planning. Therefore, besides individual professional development, training the teams that will provide services has the potential to positively affect the quality of service. In order to perform this training effectively, there is a need for innovative models in which trainees from different professions can learn from each other while making their own contributions in the learning environment.

Interprofessional education is an educational process that supports two or more professions to learn from and about each other in order to increase the cooperation between them and the quality of healthcare [17]. Team-based learning (TBL) stands out as one of the most appropriate methods for training multiprofessional teams in the interprofessional training process [18,19]. A systematic review reported that TBL is an effective method for training health professionals, with better learning, clinical, and communication outcomes, which could be used in all medical specialties [20].

TBL was developed by Dr. Larry Michaelsen in a business curriculum in the 1970s. Dr. Michaelsen defined the TBL process with three main phases as advanced preparation by learners; individual and group readiness assurance; and application, including team assignments, discussion, and feedback [19]. Peer evaluation is an additional phase of TBL [20]. Modified models using only certain TBL phases are also used [21,22]. Currently, TBL is used in undergraduate and graduate medicine, nursing, dentistry, and pharmacy programs and continuing medical education [19].

Within the scope of the abovementioned project, we planned to train multiprofessional teams that offer healthcare services for older adults under emergency conditions using the TBL method. To our knowledge, there is no study using TBL for an interprofessional education program offered for healthcare professionals working in the field of GEM.

Our aim was to develop and implement an education module for healthcare professionals engaged in GEM using TBL as the instruction method, and to evaluate the effectiveness of the program in terms of teacher and learner satisfaction and knowledge retention.

## 2 Methods

### 2.1 Participants and ethical issues

The study targeted pre-hospital and in-hospital healthcare staff who may take responsibility in emergency healthcare services for older patients. The target population was limited to personnel working in the city center of Antalya, invited to attend the training by email. A total of 324 healthcare staff (54 physicians, 98 nurses, 70 health officers, and 102 paramedics working in primary, secondary, or tertiary care settings) accepted the invitation and formed the study group by participating in the training process. Additionally, seven trainers experienced in emergency medicine and two medical education experts contributed to the study by implementing the training program and engaging with program development and evaluation.

Each participant was informed about the study in the invitation e-mails. Consent was obtained from all participants and instructors at the beginning of all survey forms. As they used their personal phones, there was no security or confidentiality problem. There were no potential conflicts of interest among the trainers or researchers.

The study proposal was reviewed and approved by the ethical committee of the University of Health Sciences, Antalya Training and Research Hospital (Approval code: 20/13/November 03, 2022).

### 2.2 Study design

**Fig. 1:**
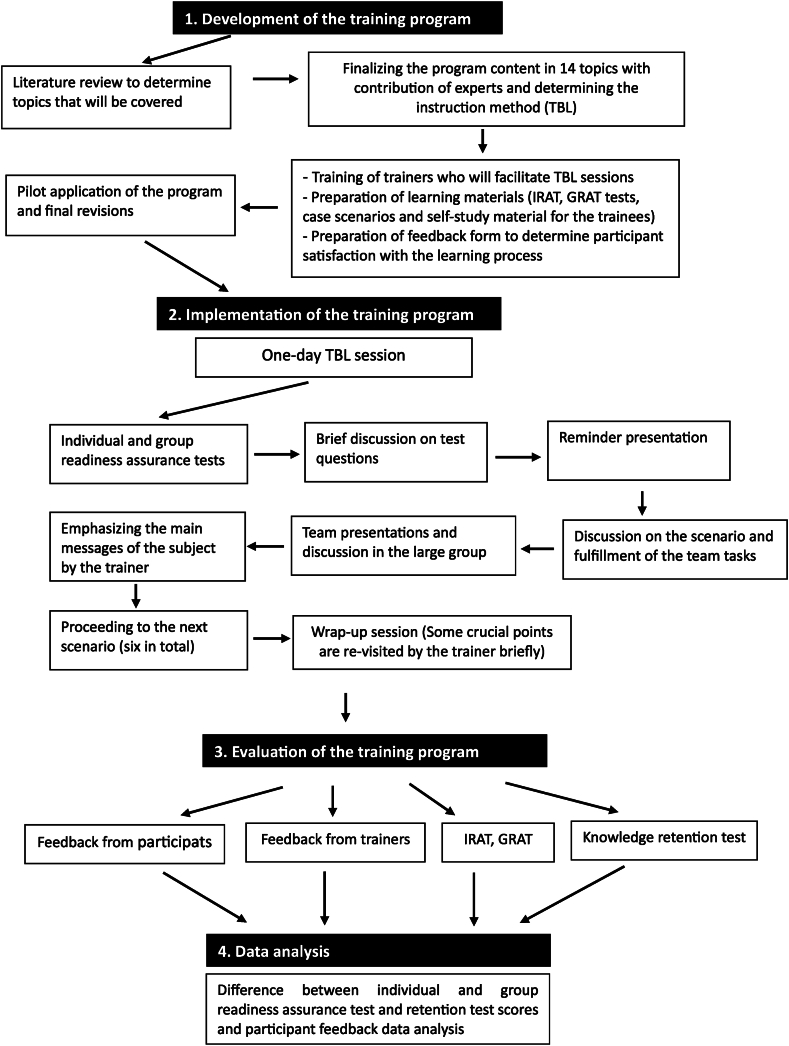
Study framework.

This was a design-based study in which a training program was developed, implemented, and evaluated. The study framework is outlined in Fig. 1 and also described in detail below.

### 2.3 Study setting

The emergency department of the hospital where the training program was implemented provides caters to more than 35,000 geriatric patients annually. This number will be twice at least when the services provided by other hospitals in the city center where the trainees work. Although GEM is a part of emergency medicine residency programs and residents are expected to gain the necessary competencies, there is no relevant subspecialty training program.

### 2.4 Training program development

The training program was designed in collaboration with the Akdeniz University Medical Education Department and Antalya Training and Research Hospital Emergency Medicine Clinic in four stages.

First, publications in the literature (textbooks, guides, and articles) were reviewed to determine which content could be covered within the program. Suggested topics were discussed between the researchers and emergency medicine specialists.

In the second stage, program content was created including the following topics and TBL was selected as the most appropriate instruction method in consultation with medical education experts.

1. Definition and physiology of aging
2. Communication with older adults both pre-hospital and in hospital settings
3. Fragility among older adults
4. Geriatric patient management in pre-hospital settings
5. Nursing care for older adults
6. Mental status changes in older adults
7. Pain management in the geriatric patient
8. Atypical clinical presentations of older cases
9. Management of life-threatening conditions in older adults
10. Trauma in older adults
11. Elder abuse and neglect
12. Polypharmacy and drug-drug interactions in older adults
13. Nursing transitions
14. Ethical and legal issues in geriatric patient resuscitation

In the third stage, the trainers attended a seminar to understand how to implement the instruction method (TBL) and which documents should be prepared before training. After the seminar, required material was prepared by the researchers and trainers. As TBL requires self-study before coming to the learning environment, relevant material including brief theoretical information on program topics and a list of some further reading resources was delivered to the participants by e-mail at least one week prior to the TBL session. Additionally, a multiple-choice question (MCQ) test including 13 MCQs was developed in the light of the literature by researchers and trainers, to be used in the individual and group readiness assurance phases of the TBL session. The MCQs are presented in Appendix 1 (https://doi.org/10.6084/m9.figshare.23895885). Finally, clinical scenarios covering all 14 program topics were created to be used in group discussions in TBL. Special attention was paid to scenarios to include commonly seen, controversial situations of older adult care, for which contributions from different professions are needed. The scenarios are presented in Appendix 2 (https://doi.org/10.6084/m9.figshare.23895885).

In the final stage of program development, a pilot TBL session was performed with small groups using prepared tests and scenarios to identify the positive and negative aspects of the process and/or material, and to make revisions if needed.

### 2.5 Training program implementation

Over a two-month period before the study, the participating trainers conducted 16 one-day training programs with trainees from various professions. An 8-h program with 20–25 healthcare professionals was implemented using a modified TBL approach each training day. The differences between the method used in this study and the classical TBL were the existence of a 1-h lecture after the readiness tests and lack of peer evaluation at the end of the training. Discussion of the readiness assurance test content and explanations of correct answers after the group readiness assurance test (GRAT) are a routine part of the classical TBL. We extended this to a 1-h lecture including not only explanations of the correct answers but also a summary of the basic concepts to be used in the team assignment phase. This was done to improve readiness levels with the expectation that it would increase learner engagement in team discussions. Although peer evaluation is a routine part of the TBL process, we did not utilize it as one day is not enough for the adequate development and observation of group dynamics.

The typical flow of the program was as follows:

After trainers and participants introduced themselves, the individual readiness assurance test (IRAT) prepared on Google Forms was administered to all participants, who used their mobile phones to answer the questions. At the top of the electronic test document, there were questions about sociodemographic characteristics and previous geriatric emergency training experiences, and how competent in geriatric emergency they considered themselves. After providing this information, the participants individually answered 13 MCQs testing their readiness level regarding basic concepts of GEM. Therefore, 20 min were allocated for the IRAT. Afterward, the participants were divided into teams of five to six, with at least one physician, one paramedic, and one nurse in each. The teams answered the same MCQs asked in the IRAT once again on a single electronic answer sheet by discussing within the group (i.e., the GRAT) in 15 min. After the GRAT process, the correct MCQ options were announced and a brief explanation was provided by the trainers for common mistakes (if any) encountered in the GRAT results. Objection of the teams to the correct answers was also considered and discussed. The readiness assurance tests and subsequent explanations and discussion took 45–50 min. Then, a trainer gave a 1-h presentation to remind the participants of the basic concepts of GEM. Although this is not a classical step of TBL implementation, regarding the concern of the trainers and emergency medicine experts that GRAT application and posttest discussions may not be adequate to ensure participants’ readiness, this section was added into the program as an extended version of the TBL phase in which the questions/answers are explained by the trainers.

The rest of the day was devoted to discussion of the six scenarios one by one and fulfilment of scenario-related team tasks. The same scenario was delivered to all teams at the same time, and they were expected to read it and discuss the content from the perspectives of the different professions represented in the team. Each scenario had some questions and/or controversial issues, and the answers of each team were determined by the joint decision of all members. Teams were allowed to use all information resources while discussing the issues and trying to find solutions. The time allocated for the teams to discuss each scenario was 30–45 min. At the end of the team discussion period, the representative of a randomly selected team presented the answers and suggested their team's solutions or dilemmas to the larger group. After this presentation, the subject was discussed comprehensively in the larger group by contribution of all teams. The other teams asked questions, added some information if needed, or mentioned alternative approaches. Trainers also joined the discussion and sometimes asked questions to reveal different perspectives. Finally, a trainer summarized the content and emphasized some main messages related to the scenario. The whole duration of handling a scenario from delivery time to the trainer's summary was 45–60 min.

At the end of the day, a wrap-up session was held in which a trainer revisited some crucial points of geriatric emergency care and gave take-home messages.

### 2.6 Training program evaluation

Program evaluation began with the readiness assurance test scores, which were automatically calculated and recorded in the online platform.

Learners’ satisfaction with the program was determined using a 17-item feedback form delivered at the end of the training day through the online platform. Items included statements about organization, infrastructure, preparation and readiness, discussion process, trainers, and general impressions. The participants were asked to access the form using their mobile phones and score each statement on a 5-point Likert scale from 1 (absolutely disagree) to 5 (absolutely agree). More than ninety percent of the study group (n = 294) completed the feedback form.

A similar 17-item feedback form scored on a 5-point Likert scale from 1 (absolutely disagree) to 5 (absolutely agree) was prepared for the trainers. All trainers completed the feedback form delivered at the end of the training process. Minimum and maximum satisfaction scores for both participants and trainers were 17 and 85, respectively. Samples of the participant and trainer feedback forms are presented in Appendix 3 (https://doi.org/10.6084/m9.figshare.23895885).

In order to evaluate the effect of the training program on long-term knowledge retention, an online MCQ test was administered to the study group six months after training using the same questions as in the readiness assurance tests. The entire study group except for two health officers took the retention test (n = 322).

### 2.7 Data analysis

Online datasets were exported to SPSS version 24.0 (IBM Corp., Armonk, NY, USA) for analysis. Descriptive statistics were used to determine the mean and median values. The difference between the mean numbers of correct answers in the IRAT and GRAT was investigated by the paired samples *t*-test. The difference between the mean numbers of correct answers in the IRAT, GRAT, and retention tests was investigated using the repeated measures test. A one-way analysis of variance (ANOVA) was used to compare the data of different professional groups and across competency levels. The Mann–Whitney *U* test was used to investigate the difference between the IRAT scores of participants who had previously received training and those who had not. P values < 0.05 were considered significant.

## 3 Results

The mean age of the participants was 34.7 ± 8.17 years and two-thirds (219/105) were women. Of the participants, 22 (6.8%) had previously attended training on GEM, 81 considered themselves fully competent, 210 felt partially competent, and 33 felt incompetent in geriatric emergency case management.

Mean scores (number of correct answers) on the 13-item IRAT and GRAT were 7.75 ± 2.48 and 11.54 ± 1.07 out of 13, respectively. The difference was statistically significant (paired samples *t*-test, p < 0.001). The mean IRAT score of physicians was higher than the mean scores of other professions (one-way ANOVA, p < 0.001; Table 1). There was no significant difference in IRAT scores between participants who had previously received training related to GEM and those who had not (8.4 ± 2.3 and 7.7 ± 2.5, respectively; Mann–Whitney *U* test, p = 0.199). Similarly, IRAT scores did not differ between groups that considered themselves competent (8.0 ± 2.5), partially competent (7.7 ± 2.5), and incompetent (7.1 ± 2.1) in GEM (one-way ANOVA, p = 0.221). The difference between IRAT and GRAT scores was significant in favor of the GRAT in every profession (paired samples *t*-test, p < 0.001 for all). However, unlike the IRAT scores, mean GRAT scores did not differ between professions (one-way ANOVA, p = 0.785) (Table 1).

**Table 1:** Mean readiness assurance test scores of different professions.

	Physician	Nurse	Paramedic	Health officer	P^a^
IRAT	9.89 ± 1.87	7.71 ± 2.34	7.28 ± 2.50	6.84 ± 2.12	<0.001
GRAT	11.70 ± 0.93	11.57 ± 1.19	11.42 ± 1.04	11.39 ± 1.27	0.785

a One-way analysis of variance.

The mean retention test score was 8.89 ± 2.80; this was higher than the IRAT mean score but lower than the GRAT mean score (repeated measures test with Bonferroni adjustment, p < 0.001), (Table 2).

**Table 2:** Readiness and retention test scores in different profession groups.

	IRAT	GRAT	Retention test	P^a^
Physician	9.89 ± 1.87	11.70 ± 0.93	10.50 ± 1.74	0.033
Nurse	7.71 ± 2.34	11.57 ± 1.19	9.55 ± 1.97	<0.001
Paramedic	7.28 ± 2.50	11.42 ± 1.04	9.54 ± 2.55	<0.001
Health officer	6.84 ± 2.12	11.39 ± 1.27	6.71 ± 3.40	0.003
Total	7.75 ± 2.48	11.54 ± 1.07	8.89 ± 2.80	<0.001

a Repeated measures test.

The mean participant satisfaction score was 81.3 ± 6.19 out of 85, and there was no difference between professions (one-way ANOVA, p = 0.748). The mean scores in the five topic areas of the feedback forms regarding participants' professions are provided in Table 3. No significant difference was observed. The mean satisfaction score was 84.6 ± 4.46 out of 85 for trainers. The comments provided by the participants were all positive. Only two participants did not consider the 1-h lecture aspect of the program particularly beneficial. However, these two participants’ total satisfaction scores were high (77 and 81 out of 85), and both of them added that if the program consisted only of lectures, they would get bored and lose their concentration. All the trainers provided positive feedback on their teaching experience. The majority of the participants and all trainers expressed their expectations about using the same method in all educational activities.

**Table 3:** Mean feedback scores by participants’ professions.

SUBJECTS	PHYSICIAN	NURSE	PARAMEDIC	HEALTH OFFICER	P^a^
Organization	4.73 ± 0.43	4.66 ± 0.47	4.71 ± 0.43	4.64 ± 0.53	0.565
Preparation/readiness	4.81 ± 0.36	4.77 ± 0.49	4.79 ± 0.38	4.72 ± 0.52	0.725
Discussions	4.85 ± 0.37	4.87 ± 0.41	4.87 ± 0.37	4.84 ± 0.40	0.372
Trainers	4.79 ± 0.44	4.79 ± 0.80	4.83 ± 0.35	4.81 ± 0.43	0.885
General	4.87 ± 0.28	4.81 ± 0.44	4.78 ± 0.48	4.68 ± 0.61	0.143
Overall	4.82 ± 0.31	4.79 ± 0.40	4.80 ± 0.33	4.72 ± 0.45	0.451

a One-way analysis of variance.

## 4 Discussion

This study was conducted with the aim of preparing, implementing, and evaluating a training program for healthcare staff on GEM. The results indicate that there is a need for such a training program, and that the program implemented in this study works well in terms of knowledge gain and retention besides learner and teacher satisfaction. The subsequent discussion is shaped around these parameters.

The need for a training program on GEM is made evident by two facts. The first is the lack of any previously implemented training program and subspecialty for GEM in the country. The second is related to our results. Although we have not found any studies indicating the existence of previously implemented training programs like ours in the country, some participants claimed that they had already participated in a training program on geriatric medicine and considered themselves competent or partially competent in this field. However, our results show that the IRAT scores of those who did not receive prior training and who did not feel competent in geriatric emergency services did not differ significantly from those who received training and who felt competent or partially competent. This finding clearly indicates that there is a need for training on GEM for healthcare staff even if they have received previous training and consider themselves competent.

We preferred TBL as the instruction method in this program, considering it suitable for interprofessional education, wherein different professional groups learn from and about each other. Our expectation was that each member would make their own contribution to improve the team's knowledge level and conceptual framework. This expectation seems to be met with the finding that although there was a difference in IRAT scores in favor of physicians, the mean scores for every profession increased and the difference between professions disappeared in the GRAT, where the team members discussed the MCQs from the perspectives of their professions. This finding clearly indicates that team members contributed to and learned from each other.

The retention test performed six months after the training program yielded scores suggesting that the knowledge levels of all professionals except health officers were higher than their initial levels but lower than the levels they achieved in the GRAT. This is an expected result as a certain degree of loss in knowledge retention is inevitable [18,[23], [24], [25]]. However, considering that there was still a difference between IRAT and retention test scores, which were based on participants' individual performances, we may conclude that the training program positively affected long-term knowledge retention. This may be attributed to the instruction method (TBL), in which the participants were actively involved with the learning process by discussing every issue in detail. The literature suggests that TBL supports long-term—but not short-term—knowledge retention [26]. This may be owing to the evaluation of short-term knowledge gain/retention generally by summative tests for which everyone prepares to attain the passing score, independent from the instruction method. When long-term retention levels are determined later through a formative test, it would be possible to reveal the real status as the test takers have not prepared for it. However, the literature contains contrary results suggesting that TBL is effective for short-term but not long-term knowledge retention [27]. This may arise from several reasons such as low levels of learner engagement with the training process, inadequate motivation of the trainees, or lack of need among some participants for such training. To minimize the knowledge retention loss in our case, we recommend planning refresher training on GEM for healthcare staff. The reason for health officers’ underperformance on the retention test compared to the IRAT may be their relatively lower participation in daily patient care practices, especially in decision making. When they have limited opportunities to use their knowledge gain, they may forget the basic principles and facts learnt in the course. This explanation would be more reliable if we had asked the participants whether they had opportunities to use the knowledge gained in the course in patient care settings. This can be accepted as one of the limitations of the study.

Another indicator to evaluate the effectiveness of the training program was learner and teacher satisfaction. We found relatively high satisfaction levels among the participants and trainers. This could be because of several reasons, such as high engagement with the learning process, participants' high motivation to learn the content, trainers’ positive approach mentioned in the feedback, or learning through solving problems commonly encountered in daily practice. The literature suggests that learner motivation and engagement is higher in TBL compared to lectures [25,26]. Additionally, the mixed nature of the training program, including the 1-h presentation besides classical TBL stages, may increase the satisfaction levels of participants who adopt different learning styles.

Postgraduate training is generally conducted with the classical method. Likewise, interprofessional training does not include innovations other than classical methods. This study is unique in that it strongly recommends the use of the TBL method in postgraduate interprofessional education as well. To our knowledge, this is the first study presenting the implementation and outcomes of an interprofessional training program in which a modified TBL method was used, in the field of GEM.

This study has some significant limitations. The first concerns the generalizability of the results. The results obtained from a single program implemented with the participation of only some healthcare staff in a local region cannot be generalized. In this regard, further studies with larger participant groups are needed to support the generalizability of the results. Another limitation is related to study design, which involved no comparisons of different methods for program implementation. Although some feedback items were available to compare the 1-h lecture and the rest of the program, we cannot be sure whether the same results would be obtained if we implemented the program using different instruction methods (e.g., lectures only). To overcome this limitation, studies comparing the effect of different instruction methods—as in undergraduate medical education—may be designed and conducted. Another limitation is that participants’ baseline knowledge was not determined before the self-study materials were delivered. If we had baseline data, we could better understand the effect of the whole training period, including prior self-study. Despite obtaining promising results regarding knowledge gain and retention, it would be valuable to assess the long-term impact of the training program on actual patient care. The current study has no data to reveal whether the knowledge gained during the program has translated into improved clinical outcomes for older patients in emergency situations. Future studies may seek information on the effect of similar training programs comparing the outcomes of patients served only by trainees with those of patients served by others. Finally, the cost may be a disadvantage for the training program because of some requirements such as training rooms, infrastructure, printed and electronic training materials, and payments to trainers and/or other staff expenses.

## 5 Conclusions

This study is unique in that it strongly recommends the use of the TBL method in postgraduate interprofessional education. We implemented a training program using a modified TBL approach to teach GEM subjects to relevant healthcare professionals with promising results in terms of knowledge gain and long-term retention, as well as learner and teacher satisfaction. This suggests that the instruction design and method used in the current study can be applied to multidisciplinary team training. We recommend that researchers who study the training of multiprofessional teams design their research considering the limitations of this study and test the effectiveness of programs using at least some of the parameters identified in this study.

## Sources of support

None.

## Funding

This research did not receive any specific grant from funding agencies in the public, commercial, or not-for-profit sectors.

## Data availability statement

Some of the data that support the findings of this study are openly available in Figshare at https://doi.org/10.6084/m9.figshare.23895885, reference number Appendix 1, 2 and 3. For the readers who asking for further data available please directly contact corresponding author via e-mail (ade.aksoy@gmail.com).

## CRediT authorship contribution statement

**Adeviyye Karaca:** Writing – review & editing, Writing – original draft, Validation, Supervision, Formal analysis, Data curation, Conceptualization. **Mustafa Daloğlu:** Writing – review & editing, Methodology, Formal analysis. **Deniz Kılıç:** Writing – review & editing. **Ramazan Sivil:** Formal analysis, Data curation. **Mustafa Keşaplı:** Writing – review & editing, Validation, Conceptualization. **Mustafa Kemal Alimoğlu:** Writing – original draft, Supervision, Methodology, Investigation, Formal analysis, Conceptualization.

## Declaration of competing interest

The authors declare that they have no known competing financial interests or personal relationships that could have appeared to influence the work reported in this paper.

## Footnotes

Appendix A Supplementary data to this article can be found online at https://doi.org/10.1016/j.heliyon.2024.e25099.
