# Supplementary material for: Training interprofessional teams in geriatric emergency medicine: A modified team-based learning approach
Source: Heliyon. 2024 Feb 7;10(4):e25099. doi: 10.1016/j.heliyon.2024.e25099 (PMC10877185; doi:10.1016/j.heliyon.2024.e25099)
Supplement: Multimedia component 1 [file mmc1.docx]

**Appendix 1. MCQs asked in readiness assurance tests and knowledge retention test**

1. What are the conditions that complicate the management, assessment, and treatment of pain in the elderly patients?

I. The development of pain due to several reasons

II. Presence of concomitant diseases

III. Increased sensitivity to drug effects, multiple drug use

IV. Lack of standard methods for assessment and treatment specific to the elderly

V. Atypical presentation

VI. Dementia, sensory disorders and disability

VII. Lack of caregivers and family support

a. I-II-IV

b. I-III-IV

c. I-II-III-V-VI-

d. I-II-III-IV-V-VI

e. All

1. A 72-year-old female patient with former diagnosis of hypertension, diabetes, and chronic obstructive pulmonary disease (COPD) submits to the emergency department. While she is being monitored with the diagnosis of COPD exacerbation in the emergency room due to the lack of any inpatient bed in the pulmonology clinic, the patient suddenly starts shouting and pulling out the vascular connections. Which of the following should be done first?

a. Physical restriction should be initiated immediately by connecting the wrists of the patient to the stretcher.

b. Haloperidol 20 mg IV should be administered.

c. The patient should be tried to calm down by explaining -with his/her relatives- why the patient is in the hospital and what has been done, together.

d. Psychiatric consultation is requested

e. Help of the security personnel is asked.

1. Which of the following is not one of the measures taken to prevent the development of delirium in the elderly patients?

a. Taking a relative near to the patient

b. Having a window in the room so that the patient can distinguish between day and night

c. Establishing a protocol in the hospital to reduce the length of stay of the patient in the emergency room.

d. Creating a noise-free environment

e. Administering prophylactic antipsychotic medication in high-risk patients

1. Which of the following is not one of the communication difficulties arising from healthcare professionals?

a. Talking too fast

b. Sudden change of the subject

c. Not providing information to the elderly patient during the intervention process

d. Empathic approach to the elderly patients

e. Not making eye contact with the patient while talking

1. Which of the following approach(es) and practices are intended to prevent communication problems with patients and their relatives during the transition to another care plan?

I- Informing and preparing the patient about where he has to submit before discharge

II. Providing adequate information to patients and their relatives about diagnosis and treatment

III. Involvement of relatives in care transition decision and selection

a. I

b. I-II

c. I-III

1. II-III
2. I-II-III
3. Which of the following is not a fragile elderly patient characteristic?

a. Low physical activity

b. Weakness

c. Weight loss

d. Hearing loss

e. Slowness

1. Which of the following statements is true for the transfer of elderly cases of falls?

a. Filling the spaces with blankets in all kinds of stretchers used for transport

b. No to empty the bag before moving the elderly patient with a urinary catheter, as it will pose a risk

c. Spinal immobilization is easier in elderly patients compared to younger patients.

d.

e.

1. Which of the following is true in terms of resuscitation ethics?

a. Resuscitation can be applied shorter for orphaned cases.

b. Resuscitation can be applied shorter for elderly patients

c. Brain death decision cannot be made in the emergency room.

d. Healthcare staff may not initiate resuscitation due to economic reasons.

e. The elderly patient is resuscitated for a minimum of 30 minutes.

1. Which of the following is not true for trauma and/or approach to trauma in elderly patients?

a. Because trauma patients need close monitoring, they should be kept in beds/stretches closest to the nurse observation area.

b. It should be considered that major trauma may develop due to reasons such as fragility, osteoporosis, sarcopenia or low-severity trauma.

c. Classical x-ray should be preferred as the imaging method, and tomography should be avoided due to radiation load.

d. The main causes of trauma are age-related changes such as decreased vision and hearing, weakness in the musculoskeletal and nervous system, impaired reflexes and/or slowed reaction time.

e. For health personnel, the main findings suggesting that the patient has been abused are poor hygiene and nutrition, delayed post-traumatic hospital admission, inconsistency in trauma history, or dominant behaviors of the elderly relative.

1. Which of the following statements about aging is wrong?

a. Human metabolism slows down with age

b. Orthostatic hypotension frequency increases with age.

c. Frequency of urinary tract infections increases after menopause in women

d. Glomerular filtration rate increases due to decreased kidney mass

e. The response to bleeding and hypoxia decreases due to the decrease in bone marrow mass and stem cell count.

1. Which of the following is incorrect in polypharmacy and its management in elderly patients?

a. Polypharmacy is the simultaneous use of more than one drug and is generally defined as the use of more than 4-5 drugs together.

b. Drug interactions related to polypharmacy may lead to situations such as falls, changes in consciousness, functional regression and weight loss.

c. Medications most frequently misused by elderly patients are warfarin, insulin, antiplatelets and oral antidiabetics.

d. In the presence of hypertension and chronic renal failure, the use of non-steroidal anti-inflammatory drugs is safe.

e. In order to prevent fragility in the elderly patient, the use of multiple drugs should be reduced.

1. Which is wrong in Cardiopulmonary Resuscitation (CPR) applications in the elderly patient?

a. In the absence of teeth, mask ventilation and intubation may be difficult.

b. Decreased upper airway muscle tone and loose lip structure not supported by teeth make airway management more difficult.

c. Decreased elasticity of the chest wall makes ventilation with balloon-valve-mask difficult.

d. It is recommended to reduce the drug doses used in fast serial intubation by 30-50%.

e. Compression depth should be reduced in order not to damage the ribs during chest compression.

1. Which of the following is/are true about elderly patients presenting with atypical complaints/symptoms?
2. In 20-30% of cases with infection, fever, leukocytosis and elevated CRP may not be present.
3. Delirium may be the first or only symptom of a serious and life-threatening condition such as sepsis, pneumonia, or myocardial infarction.
4. Acute coronary syndrome is not considered in the patient without chest pain.
5. Defense and rebound may not be detected in the abdominal examination even if a serious pathology underlies.
6. Abdominal aortic aneurysm/rupture should be considered first in the presence of hypotension, abdominal pain and a pulsatile mass in addition to back pain.
7. 30% of abdominal aortic aneurysm cases are clinically confused with renal colic.
8. Especially in the presence of sudden onset of new behavioral and psychiatric symptoms, dementia should be considered first.

a. I-II-VII

b. I-V-VII

c. II-III-V-VI

d. I-II-IV-V-VI

1. All
