# Supplementary material for: Training interprofessional teams in geriatric emergency medicine: A modified team-based learning approach
Source: Heliyon. 2024 Feb 7;10(4):e25099. doi: 10.1016/j.heliyon.2024.e25099 (PMC10877185; doi:10.1016/j.heliyon.2024.e25099)
Supplement: Multimedia component 2 [file mmc2.docx]

**Appendix 2. Scenarios used in team discussion sessions**

1. **COUMADIN OVERDOSE**

**Learning Objectives**

**At the end of this session the participants are expected to:**

1. Manage unstable patient in the field and during transfer

2. List the nursing care services to be applied to patients with low Glaskow Coma Scale?

3. Correlate the drugs used by the patient and his/her history (herb food and coumadin use)

4. Consider the causes of mental state disorders in the differential diagnosis (Central Nerve System infections, meningitis, agitating causes, etc.)

**CASE INFORMATION**

An 84-year-old female patient is brought to the emergency room by 112 Emergency Health personnel with the complaints of sudden onset of headache, nausea, vomiting and deterioration in consciousness. To the patient, who continues to vomit actively, was given fluid in the ambulance and 1 ampul metochlorpromide was administered. The signs of incontinence observed.

They say he suddenly had a headache while sitting next to his neighbor, he started to vomit and had difficulty in speaking. The next-door neighbor states to the 112 teams that the patient attended his neighbor's wedding dinner and ate a pot of herbs cooked in the cauldron 3 days ago, and that he felt a bit tired and sluggish after coming back from the wedding.

History: DM for 30 years-Using Oral Antidiabetic-Blood sugar is generally regulated, Hypertension-ACE inhibitor is generally regulated for 20 years, Mitral Valve replacement-Coumadin 5 mg -5 years-generally regulated. He did not recommend it, he was discharged saying that everything is okay.

Background: No features

The general condition of the patient is moderate, unconscious, responds to painful stimuli. GCS:E3M4V4:11.

Vital Findings: TA;190/110 mmHg, Respiratory rate: 22/min, Fever:36.5C, heart rate:130/min, rhythmic, Pulse OXimeter:96, in room air

As a positive finding in physical examination; The skin is moist, the pupils are anisochoric, the eyes are left lateralized, the light reflex is present bilaterally, there are crepitant rales at the base of the lung.

There is no obvious pathology in other system examination findings.

Patient’s cranial CT shows 5-7 cm intracranial hemorrhage and he hospitalized in the intensive care unit after Neurosurgery and Neurology consultations.

QUESTIONS:

*1. What should be considered in the management of the patient, starting from the scene and in the pre-hospital process?*

*2. How should the management be in the emergency service?*

*3. What are the points to be considered in terms of nursing care?*

*monitor state of consciousness*

Follow with monitor

**ASSESS THE RISK OF FALLING**

Identify risk of falling install warning signs

Always keep the hospital bed at its lowest level

Respond to the emergency call system as soon as possible

Use bed borders when needed

Go to patient restraint procedure if necessary.

Eliminate stimuli, inhibit agitation

Put elderly’s bed with a high risk of falling in the room close to the nurse's desk, where you can see them if possible.

In order to minimize the side effects (hypoglycemia, etc.) of drugs that may cause falls, follow up frequently and report deviations to the physician,

**- Aspiration risk:**

Dentures come off

Raise the head of the bed to 45 degrees.

Watch the elder while swallowing/swallowing

Evaluate for abnormal respiratory sounds (rales, rhonchi, wheezing, etc.), dyspnea, cyanosis, hyperthermia, and worsening of consciousness.

Assess the intensity of intraoral secretion.

Have the aspiration system readily checked

**-Risk of deterioration of skin integrity:**

Watch the areas with skin discoloration, don’t lay on it, change positions frequently.

Support bony prominences with protective materials that prevent compression

Change positions at least every two hours

Use assistive devices and multiple staff during position change

**-Infection risk:**

Pay attention to aseptic application of urinary catheter, evaluate daily for discharge, redness, color of urine

Monitor for signs and symptoms of infection (body temperature, pulse rate, respiratory sounds, rales, secretion, appearance of urine, skin temperature, etc.)

Evaluate the factors that increase susceptibility to infection (advanced age, suppression of the immune system, malnutrition, etc.)

Monitor laboratory findings (CBC, granulocyte count, blood and urine culture, serum albumin and protein values, etc.)

Evaluate the skin for tears, abrasions, redness, or ulcers

**- Impairment in physical mobility:**

Teach proper position and movement

Provide the possibility of using a trapezoidal bar in bed

Encourage for help

Follow, record and evaluate physiological needs such as sleep and nutrition.

Put the objects that will be used frequently in easily accessible places.

Educate the patient/patient relatives about drug/drug, food/drug interactions at discharge

*4. What are the etiological factors that may cause the patient to fall into this situation?*

1. **SUBDURAL HEMORRHAGE**

**Learning Objectives**

**At the end of this session the participants are expected to:**

1.Comprehend that elderly patients commonly submit to the emergency department with atypical clinical presentations

2. Be aware that there may be serious pathologies underlying atypical clinical presentations.

3. Apply evidence-based medicine principles while selecting required diagnostic tests in elderly

4. Take detailed medical history and relate the findings (especially former diagnoses) with the current symptoms of the elderly patient

5. Recognize and appreciate the importance of nursing care services (close monitoring, appropriate environment etc.) for the elderly patients who submit with atypical clinical presentations.

**CASE INFORMATION**

A 78-year-old male patient with complaints of fatigue and weakness is brought to the emergency room by the 112 ambulance. The 112 ambulatory team, who transferred the patient gets information from the caregiver visiting the patient once a week to meet his basic needs. The patient lives alone. His current complaints have been present for 5-6 months with complaint-free periods. However, the frequency and duration of complaints have increased recently. The patient has no additional complaint.

**Past medical history:** Hypertension and diabetes mellitus for 20 years.

Regular medications: Delix plus 10 mg tb, 1 x 1, Metformin 1000 mg tb, 2 x 1, Coraspin 100 mg tb, 1 x 1, Vit D –Colefor 20.000 ü tb 1 x 1.

The patient fell at home 3 months ago and submitted to emergency department. After examination, tomography and follow-up in the emergency room he was discharged.

**Family history:** Lung cancer and hypertension diagnosis of his father

**Physical examination**

The general condition of the patient is good to moderate, he is conscious, oriented, cooperative, and prone to sleep from time to time.

TA:150/80 mmHg, Heart rate: 78/min, rhythmic, PulseO2: 95, Body temperature :36,7 C , GCS=15

Oral mucous membranes are dry

Crepitant rales (crackles) are heard in bilateral lung bases.

There is + pitting edema in the lower extremities.

Blood sugar =137 mg/dl (measured by stick)

ECG= Atrial fibrillation

**QUESTIONS**

Which laboratory tests are required for this patient?

Is imaging needed for this patient?

Which pathologies should be considered prominent in differential diagnosis?

What is the treatment approach/plan?

1. What are the points that should be especially considered in the management of the patient in the pre-hospital process, starting from the first contact?

2. How should the management of the case be in the emergency department? Which diseases should be considered in the differential diagnosis?

3. What are the points to be considered in terms of nursing care?

4. Which factors as the causes of falls/syncope should be evaluated?

**3. SYNCOPE**

**LEARNING OBJECTIVES**

**At the end of this session the participants are expected to:**

1. Be aware of the possibility of major injury following low-energy trauma

2. Explain pre-hospital management features of the patient with major trauma

3. Recognize the nursing care services of the patient with major trauma in the emergency room

4. Be careful about the drugs used by the patient and possible complications (Alpha 1 blocker, etc.)

5. Distinguish the causes of falls as traumatic and non-traumatic and exclude life-threatening conditions (ACS, PTE, Aorta dissection, aneurysm rupture, hypovolemia, cardiac conduction disorders)

66 years old, male, patient has been brought to the emergency room by 112 due to an incision in the forehead. A cervical collar was attached to the patient by 112 ambulance personnel. The patient was walked to the ambulance because he wanted to walk. When the teams reached the patient, the patient was agitated. That's why they said they couldn't implement a vascular access, they couldn't measure the vital signs. The patient is taken from the resuscitation area to the surgical yellow area. When the patient was asked about the trauma mechanism, he says, “I got out of bed and headed towards the toilet. I felt dizzy, my foot got stuck on the carpet. I don't remember what happened next”. Relatives of the patients say that there is no carpet in the corridor. Patient says she had nausea before she fell down. He hit his head on the corner of the wall in the hallway. When his relatives heard the voice, they rushed to him. He had some urinary incontinence. He does not describe stool incontinence. He has DM, HT comorbidities. He says he had a cataract operation 2 years ago and it was a bit of a problem. He uses his medications regularly and does not interrupt his follow-ups. He says he just came for a checkup two days ago. Medication has been adjusted. He doesn't remember which medication they changed. Medicines are not with the patient. In the medicine bag; there are Glifix 30 mg tb, Diamicron MR tb, Ex-forge 10/160mg tb, Doxura 2 mg tb.

On physical examination, there is a 3 cm subcutaneous incision on the left half of the forehead with irregular margins. There is edema under and around the incision. Pupils are anisocoric. Left pupil is irregular, light reflex cannot be obtained. There is no oedema, no hyperemia. There is no cervical tenderness. There is no feature in other system examinations.

TA: 110/60 mmHg, Pulse: 65/dk spO2:%96 Blood glucose: 120mg/dL

What do you think, what would you like to do?

The patient's ECG has NSR, a rate of 66/min, no ST-T wave change. In the brain CT, there is no feature other than subcutaneous soft tissue swelling in the left frontal area.

Hb: 14.2 WBC: 12000 PLT:350000 Glu:126mg/dL BUN:18 Crea:1.1

- The patient's incision was sutured in the area. Tetanus vaccine was injected because she did not remember the tetanus vaccine.
- - The patient stated that he had severe pain. The physician ordered 5 mg morphine IV slow push to relieve the patient's pain.
- - The relatives of the patients run towards the desk saying that their patient has fainted again. When you went to the patient, you saw a deviated eye and a couple of clonic jerks. The clonus then stopped, but the patient has wheezing and unconscious.

What happened now? What should we do?

For the narrator:

112 PROCESS:

Even if the patient does not want it, a trauma board should be used because the mechanism of trauma is unknown.

Although there are sometimes problems in agitated patients, vascular access should be implemented.

Patients must be in the security circle in the ambulance (vital signs check, vascular access, monitoring)

NURSING PROCESS:

- Fall risk:

o Assess fall risk

o Follow the patient closely

o Choose the bed to consider

o Evaluate orthostatic hypotension/report deviations to physician.

o Respond to the emergency call system as soon as possible

Fall risk: Assess fall risk.

- Identify the fall risk, install warning signs

- Put auxiliary tools within reach

- Keep the hospital bed at the lowest level all times

- Answer the emergency call system as soon as possible

- Use bed borders when needed

- Orient the elderly individual to the environment

- Make sure that the patient's room is well lit, use nightlights at the bedside if necessary.

- Teach how to move using safety measures such as grip

- Put elderly’s bed with a high risk of falling in a room close to the nurse's desk, where you can see them if possible.

- In order to minimize the side effects (hypoglycemia, etc.) of drugs that may cause falls, follow up frequently and report deviations to the physician,

- -Infection Risk:

- Pay attention to aseptic application of possible urinary catheter, evaluate discharge, redness, color in urine Daily.

- Monitor signs and symptoms of infection (body temperature, pulse rate, respiratory sounds, rales, secretion, appearance of urine, skin temperature, etc.)

- Evaluate the factors that increase susceptibility to infection (advanced age, suppression of the immune system, malnutrition, etc.)

- Monitor laboratory findings (CBC, granulocyte count, blood and urine culture, serum albumin and protein values, etc.)

- Assess the skin for tearing, scraping, redness or ulceration

- IMPROVEMENT IN PHYSICAL MOBILITY:

- Teach him to take proper position and move

- Provide the possibility of using trapezoidal bars on the bed

- Encourage asking for help

- Follow, record and evaluate physiological needs such as sleep and nutrition.

- Put the objects that will be used frequently in easily accessible places.

- RISK OF SKIN INTEGRITY:

- Watch the areas with skin discoloration, don’t lay on it, change positions frequently.

- Support bone protrusions with protective materials that prevent compression

- Change positions at least every two hours

- Use assistive vehicles and multiple personnel during position change

MEDICAL PROCESS:

Doksura was started in the patient's control visit 2 days ago. The physician who started the drug did not warn the patient about the first dose hypotension. When viewed from the national health system, it turns out that the drug has just started. The cause of the patient's urinary incontinence is overflow incontinence.

His pupils were damaged during the cataract operation, and the light reflex could not be obtained for this reason.

If the patient was suspected of orthostatic hypotension, a significant difference in blood pressure would have occurred.

Morphine administered to the patient, who was already hypotensive for pain and could not cope with this condition, deepened hypotension and caused syncope to develop again. Jerks and audible breathing were due to hypotension and were caused by transient cerebral ischemia. After the patient's hypotension is corrected and the emergency observation period expires, the first dose of hypotension can be explained and the patient can be discharged from the emergency room.

1. **NEGLECT AND ABUSE**

**Learning objectives:**

**At the end of this session the participants are expected to:**

1. Notify the judicial authorities when there is suspicion about neglect and abuse (inconsistent points in the history, history that does not match with the physical examination, failure to follow up in the outpatient clinic, etc.).
2. Perform a complete physical examination rather than a focused one in elderly
3. Use appropriate language when talking to the patient and his/her relatives, get and give information by empathizing general expressions.

Time: 11:45

You're wanted from triage.

The triage officer asks you: There is a patient in the triage. Relatives causing trouble. She says that his patient is old, hit her head, and may be suffering from a cerebral hemorrhage, and she asked the doctor to examine his mother without waiting. What should we do?

What's wrong with the patient? you ask.

76 years old female patient

Complaint: Falling. Laceration on the scalp. GCS:15

Vitals: BP:135/82 mmHg, Pulse: 112/min, Fever: 36.3 C^o^, O_2_Saturation: 99%

Resume:

• Diseases: AF+ CVD(right hemiparesis+, 4/5)

• Medications: coumadin, diltizem

• She can walk inside the house with a walker.

You tell the triage officer to come.

The patient's relative (PR) brings the patient into the room in a wheelchair. PR is angry. “You were finally able to look at us. And you called this place the emergency room.” she complains.

You ask the patient what is the problem with her. The patient's relative answers.

PR: After taking a bath, my mother slipped and hit her head. As you can see, she has an incision on his forehead.

You:Did you hurt any other part of your body?

PR: No, she didn't, thank God she didn't fall hard.

You: How did you fall? Did you have fainting, chest pain, or headache prior to falling?

PR: I just told you that while getting up from the bathtub, her foot slipped.

You: Please let me talk to your mother

PR: My mother has already hit her head. We waited a long time until she came to you. Let's do a brain tomography and see if there is any bleeding in the brain, let's sew the incision and let us go home without tiring my mother out even more.

You: Let's examine first and ask the patient to get on the stretcher.

You saw that she had a limp on her left hip as she was getting up from the wheelchair to the stretcher. In the meantime, you noticed a sourness on the patient's face.

You: Does your hip hurt?

Patient: Yes

You: How long has it been kept going? Were there a falling or crash?

PR: My mother tripped and fell on the carpet last week. Her eyes can no longer see clearly, you know, old age. Mom, how many times have we talked about not walking around without wearing glasses?

(His mother looks down guiltily.)

You: Did you go to the hospital and have it checked?

Patient: No, I don't need to, I can walk.

Physical examination: There is an incision of approximately 2 cm on the right side of the forehead. There is a slight hematoma underneath but not large. No active bleeding. You do not feel a stepping or crepitation underneath on palpation. There is yellow abrasion in a small area laterally on the neck.

Neurological examination: GCS:15 light reflexes are complete, pupils are isochoric, and eye movements are complete. You do not detect any new abnormality except right 4/5 hemiparesis (old sequelae).

Pelvic examination of left hip movements was painful. You did not see any deformities.

Examinations:

ECG: AF+ 111/min

Hb: 11.5

Plt: 160 000

ALT/AST: 25/30 BUN: 12 Cre: 0.6

Electrolytes: within normal range

INR: 0.9

Brain CT: normal

Pelvic CT: non-displaced left pubic inferior ramus fracture was detected.

Intervention: You planned tetanus prophylaxis + suture + dressing and orthopedic consultation.

The orthopedic doctor came to the emergency room for another patient. After evaluating your patient, he orthopedically recommended bed rest, prophylactic cleaxane if not contraindicated, and orthopedic outpatient control. You guided the patient and their relatives to the surgical area with the personnel for suturing.

While the medical officer is making preparations in the surgical area, the patient's relative accidentally spills the water that his mother gave him to drink on the patient. While the patient's relative was trying to quickly remove the blouse before it got wet, the health officer noticed the large ecchymosis on the patient's back when the undershirt was peeled off for a short time.

The attendant tells the patient's relative that he can buy clothes from the canteen, and when the patient's relative leaves, he immediately informs his doctor. The hospital security is also informed and told to delay if the patient's relative returns. History taken from the patient when the patient is alone:

Patient: A week ago she asked her daughter to bring water because her walker was not near. Her daughter said that she was too tired and she couldn't bring it now. Patient told that her daughter works hard and she is a burden to her. She tried to stand up on her own, but lost her balance and fell on the stair step next to the seat. She hit her back on the step. She felt so much pain that she wanted to go to the hospital. However, her daughter tried to get the patient to her feet and did not take her to the hospital, saying, "Look, it's okay, you can walk, if necessary, we'll go to the polyclinic" when she could stand up. The patient also tried to endure the pain and gave his daughter the right.

This time she asked for help to get out of the tub while taking a bath. Her daughter said she was coming too, but when she didn't come even though she waited for 10 minutes, she got cold and tried to get out on her own. She lost her balance and hit her head on the tile. She called her daughter. Then they came to the emergency room.

She says that her daughter is actually a good person, there are occasional small arguments, but she thinks that her daughter is very tired and she gives her the right. The patient also thinks that she is a burden to her daughter. She asks you not to tell her daughter about this. She says she has no other ministers, doesn't want to live in a nursing home, is dependent on her daughter, doesn't know what to do without her, so she doesn't want to piss her off.

The anamnesis taken from the patient's relatives later: She tells thet her other two siblings did not help at all for the care of her mother, she has to work and earn money and take care of her mother, her living conditions are difficult, she could not pay the rent, she could not bear it anymore, she could not meet her mother's needs, and she only wants to sleep when she comes home.

Tasks:

1. What caught your attention? What were the indicators of abuse and/or neglect?

2. What was missing or done wrong?

3. What should be considered in the nursing approach for this patient?

4. What role can healthcare professionals play in revealing such situations?

5. If it is thought that there is abuse/neglect, how to talk to patients and their relatives, what should be asked?

Answers:

1. Indicators of abuse and/or neglect:

• In the first incident (the incident one week before the application), the patient's relative did not respond to a basic need such as water for the patient, allowed her to take water alone when the walker was not present/neglected.

• Again, in the first fall, the patient was not brought to the hospital and checked / neglected

• In the second event (at the time of admission in our case), the patient’s relative did not respond to the patient's call even though she called her relative / neglected.

• The ineffectiveness of the drug blood level indicates insufficient follow-up/ neglected.

• The patient's relative does not allow the patient to talk. She is trying to advance the dialogue within the framework of her own story/trying to hide her negligence.

• She tries to show that she cares about her patient by making a scene in triage, and trying to reflect the blame on healthcare professionals.

2. When the doctor went to the physical examination after the anamnesis, he detected a finding that was not mentioned by the patient. (left flank limp and pain). Considering the estimated prevalence of neglect/abuse in elderly patients as high as 5-10%, this patient should have had a complete physical examination rather than a focused one. Fortunately, if the patient's clothes had not been stripped in the surgical area, the ecchymosis on the patient's back would not have been seen. This finding would have been detected had a complete physical examination been performed.

The patient's relative was constantly confused while taking the story from the patient. She did not allow the patient to speak. This is not a normal behavior. The patient's relative should be warned politely a few times, and if this behavior still continues, the patient and his/her relative should be separated from each other by agreeing with the staff and sending the patient's relative to a record acceptance or another place, and both parties should be interviewed one by one. If the staff in the surgical field had not warned the doctor, the doctor would not have made the individual interviews. Doctor warned the patient's relative only once and at one point he accepted the involvement of the patient's relative in the anamnesis.

Since this patient uses coumadin and the history is not reliable (the patient's relatives are constantly confused about the story), it is appropriate to be examined and ECG taken since the differential diagnosis of syncope is made. (I added this paragraph to answer anyone who asks why the patient was examined)

**3. Nursing approaches:**

**Falling risk:**

- Assess the risk

- Evaluate orthostatic hypotension

- Recommend assist vehicles to stay within reach

- Orient the elderly individual to the environment.

- Make sure that the family / relatives stay with the elderly, if there are glasses and hearing aids, make sure that they are used.

- Teach you how to move using safety measures such as grip

- If necessary, accompany the elderly during mobilization, teach how to

- Take the elderly person who is at risk of falling to a room close to the nurse's desk.

- Have him use a walking aid if necessary.

- Teach you how to fall to minimize injuries

**Risk of injury:**

- Evaluate vital signs

- Put the devices he routinely uses in an easily accessible place

- Make sure to bring important items for the elderly

- Check every 30 minutes

- Apply your medications according to the doctor's order

**Risk of infection:**

- Follow the individual hygiene practices against infection and give the necessary training to the patient / patient's relative

- Inform about wound care and infection rules

**Inability to cope with family:**

- Identify suspected elder abuse cases, observe/evaluate the following findings.

o Failure to comply with treatment regimens.

o Findings of malnutrition, dehydration

o Wounds, bruises, swelling, lacerations, burns, bite/teeth marks

o Pressure ulcers

o The caregiver does not allow the elderly to stay with the nurse.

- In doubtful cases (Anetzberger, 1987):

Know the laws in your country, region on elder abuse.

Provide objective recording, including:

o Identification of traumas

o Conversations with the elderly and caregiver

o Defining behaviors

o Nutrition, hydration status

Support the capacity to make choices. Consider the right of the elderly to choose to live at risk of harm.

Do not take any action that may increase the risk of harming the elderly or provoke the abuser.

-Respect the elder's privacy and free will

- Inform the society on prevention/protection.

4. **112:** The home environment of the patient, if the patient is bedridden, the condition of the environment where the patient is cared for, the attitude of the relatives of the patient towards the patient at home can be informative in terms of neglect and abuse. When talking about the patient, if the relatives of the patient use objective pronouns such as this, or if the patient has long fingernails, dental problems, or if the patient smells bad, neglect should be considered. It is very important to inform the doctor to whom he is delivered in the emergency about this issue. The findings of the 112 team may sometimes be the only clues. When information is received in this way, the physician should not make statements that will leave 112 in a difficult situation in the presence of the patient's relatives as much as possible, and should make an effort to catch their own clues in the light of this information. It should not be forgotten that the physician is obliged to suspect and report. He does not have to prove his suspicion. If possible, separate information should be obtained from the patient and their relatives, and the patient should be allowed to express himself. This can sometimes be time consuming. However, the moment the patient is brought to the emergency room may be the only time the patient has contact with the outside. This opportunity is worth considering. A careful anamnesis should be taken, if a physical examination that does not match the anamnesis is detected, abuse and neglect should be suspected and the judicial authorities should be informed.

Hospital staff: should pay attention to the behavior of the patient's relative to the patient while taking the patient for imaging, rude indifferent attitudes, do not talk to the doctor about this, why you said this, should inform the patient's doctor about this. In the studies, it has been observed that the relatives of the patients talk freely among themselves and exhibit suspicious attitudes, especially during imaging, and this situation is determined by the hospital staff or radiology technician.

In short, all personnel working in the 112 and emergency services should inform the patient's physician if they suspect negligence and abuse.

1. The moment of application to the emergency service of a patient who is a victim of neglect and abuse may be the only time to identify these patients. This should be seen as an opportunity. Physically, sexually, psychologically, financially, patients can be abused or neglected. If possible, a complete physical examination should be performed. Is there ecchymosis on the wrist and ankle? (restrictive used), does the story told match the physical examination? (protected by falling; no trauma is expected on the inner surface of the thigh, arm and forearm). If possible, try to separate the patient and their relatives. Try to learn the story from both. If there are inconsistent points or inconsistent physical examination findings during the cross-examination, try to speak without frightening the patient and his relatives. While talking to the patient, make inquiries about general questions and abuse and neglect without being judgmental and accusatory. Do you have moments at home where it hurts, instead of saying it hits you? Do you sometimes have to do something you don't want to do? It is suggested to ask general questions such as whether you are asked to sign some papers. When talking to the patient's relative: Do not be accusatory, try to get information from the patient's relatives with a sweet language by empathizing. If you are directly accusatory, they will become aggressive towards you and your dialogue will end. According to the Turkish Penal Code, if abuse and negligence are suspected, it is a judicial duty to notify the legal authorities of this situation by the physician and to keep the judicial file. Find out what your patient thinks about this situation after this task has been accomplished. Try to find out if he has the ability to decide if he still wants to return to the same house and to the abuser. Psychiatric consultation may be appropriate. If he has the ability, respect the patient's wishes (let's emphasize again, even if the patient says not to report it to the police, we are obliged to report this point separately). If the decision-making capacity is not sufficient, additional information should be given to the social services and the prosecutor's office.
2. **ELDERLY PATIENT WITH FALLING HISTORY (NURSING CARE)**

**Learning objectives:**

**At the end of this session the participants are expected to:**

1. List the main components of nursing care
2. Explain the algorithm that should be applied to protect skin integrity
3. Explain the basic elements of communication with the elderly patient
4. Analyze the risk of falling in the elderly patient

**CASE INFORMATION:**

An 82-year-old male patient is brought to the emergency room by 112 with the complaint of falling from his own level at home. The patient was removed from Korkuteli with the decision of Askom.

The transfer of the patient took approximately 45 minutes on the trauma board. During the first examination and referral process in Korkuteli state hospital, he was hospitalized on the trauma board for 45 minutes. Again, he is taken to the examination room with a trauma board.

No explanation was given to the patient in Korkuteli or on the road. The patient is experiencing severe emotional stress.

112 personnel did not take the patient's hearing aid and glasses with them.

As the patient cannot hear and see clearly what is going on around him, his stress increases.

The trauma board presses on the bony prominences and this creates pain. In addition, the patient is insisted to lie still on his back.

There is no clock around the patient, time disorientation is broken. No legible signage stating where it is. Ground orientation is broken.

The emergency room environment is very noisy. The patient is not with him. The light level is too high.

The patient wants to express his severe pain but cannot reach the help button. Orientation information about the environment was not given to the patient. He doesn't know where the napkin, water and other belongings are.

The patient was stripped for examination upon arrival, but only 1 sheet was covered afterwards. The patient feels cold and the risk of hypothermia develops.

While the doctor starts the consciousness examination by saying how are you, the staff begins to undress the patient, one of the nurses starts to open an intravenous line and the other starts to measure vital signs. Because the patient has scoliosis, he is uncomfortable lying on his back, but the patient is not allowed to move and analgesics are not administered.

The patient is again sent to the radiology department for imaging on the trauma board. Examination and analysis results, consultations, etc. procedures continue while they are waiting on the trauma board, and no interventions are made to reduce the pressure on bone protrusions. On top of that, the patient is fixed on the bed because he is constantly moving due to his discomfort.

The doctor and nurse do not give him any information about the patient's condition and the process. Since the lying position does not change every 2 hours, color changes begin in the areas exposed to pressure. With the movement of the patient, superficial bleeding begins in some skin areas whose blood supply is impaired.

In this process, the patient does not insert a bladder catheter and the patient wets the bed.

Although about 4 hours have passed since the process started in Korkuteli, the patient is not given water and food, and fluid therapy is not started to prevent dehydration. The patient is at risk of dehydration and hypoglycemia.

The patient goes into delirium.

The patient, who goes into delirium, somehow dissolves the fixation materials and falls out of bed.

The fall risk of the patient was not evaluated and a fall wristband was not worn due to the high risk of falling.

There is no mechanism around that the patient can hold on to and get up from.

Since the patient's bed is in a far corner from the nurse and doctor desk, it is noticed late.

The patient needs resuscitation, but the resuscitation car is in a remote location.

**QUESTIONS:**

1. What are the mistakes you observed in the patient's referral process and emergency service management?

2. Are these mistakes made frequently in your daily practice?

**EVALUATION:**

1. Be prepared for transport

2. Hemodynamic monitoring, securing the airway

3. Fluid intake and output (MLST)

4. Observing the effects and side effects of drug and fluid infusions

5. Patient safety despite the risk of falling/trauma due to the drugs used

6. Relatives of the patient can be taken with the patient with Alzheimer's and dementia.

7. Materials and medicines must be ready and in working condition

8. The physician should be informed when there is a sudden hemodynamic change or a change in the character of the symptoms.

**GENERAL EVALUATION:**

1. Risk of Deterioration of Skin Integrity

2. Risk of Falling

3. Disruption in gas exchange

4. Constipation

5. Stress Incontinence

6. Risk of Injury

7. Risk of Infection

8. Impaired Physical Mobility

9. Risk of Ineffective Tissue Perfusion

10. Hypothermia

11. Sleep Pattern Disruption

12. Risk of aspiration

13. Risk of Fluid Volume Deficiency

For further reading;

**1. Risk of Deterioration of Skin Integrity:**

- Identify the factors that increase the risk of deterioration of skin integrity

- Identify sources of pressure and friction that may cause trauma to the skin

- Observe skin under pressure during repositioning or movement

- Assess the skin by observing and touching from head to toe twice a day

- Evaluate endotracheal tube, nasogastric tube, and tracheostomy cannula fixation sites

- Evaluate the skin for non-fading redness, localized warmth, edema, acne, vesicles, burns, rashes, swelling and bruises in areas that touch the bed.

- When cleaning the skin, avoid using soap, alcohol and hot water with irritating acidity, clean with warm water (32.2 -40.5 0C) and use a moisturizer.

- Clean the older person's face, axilla and genital area as needed.

- Laying on the area with skin discoloration.

- Make sure wet areas (perineum and sacral) are clean and dry.

- Provide adequate and balanced nutrition, cooperate with a dietitian if necessary

- Support bony prominences with protective materials

- Maintain adequate fluid intake to ensure adequate hydration

- Raise the head of the bed by more than 300C to reduce folding, shearing and pressure

- Avoid rubbing or massaging the areas with redness and bony prominences.

- Change positions at least every two hours

- Use assistive tools during position change

**2. Risk of Falling:**

- Identify individual and environmental factors that increase the risk of falling

- Assess the risk of falling

- Wear a wristband that indicates the risk of falling

- Put auxiliary tools within reach

- Review the hospital's "patient transfer" protocol

- Keep the hospital bed at the lowest level at all times

- Answer the emergency call system as soon as possible

- Use bed borders when needed

- Recommend to wear shoes or slippers with non-slip soles

- Orient the elderly individual to the environment

- Avoid rearranging items in the room

- Make sure the patient's room is well lit, use a nightlight at the bedside if necessary

- Make the family/relative stay with the elder

- If you have glasses and hearing aids, make sure they are used

- Teach you how to move using safety measures such as grip

- Encourage him to participate in a regular exercise and gait training program

- Accompany the elderly during his mobilization if necessary

- Put the elderly at high risk of falling in a room close to the nurse's desk

- Have him use an assistive device (cane, walker, etc.) to walk if necessary

- Provide assistive devices for transfer and ambulation of the elderly, if needed

- In order to minimize the side effects (hypoglycemia, etc.) of drugs that cause falls, report the situation to the Physician,

- Teach you how to fall to minimize injury

**3. Disruption in gas exchange:**

- Monitor and record the respiratory rate and depth, respiratory sounds, cough, sputum and mental status of the elderly regularly during and after admission to the ED.

- Assess for changes in the behavior or mental state of the elderly, such as anxiety, apathy, hostility, and restlessness.

- Check oxygen level (keep it greater than 92%) or review arterial blood gas findings (optimal PaO2 80% - 95% or higher).

- Encourage breathing and coughing exercises.

- Encourage the use of spirometry.

- Encourage fluid intake (more than 2.5 liters per day) if not contraindicated.

- Prevent hyperthermia, reduce pain and anxiety.

- Teach them to use assistive devices such as nasal cannula or oxygen mask.

**4. Constipation:**

- Evaluate and record the normal bowel habit of the elderly individual.

- Assess hydration status.

- Identify factors that can cause constipation

- Explain to the elderly the relationship between fluid intake and constipation.

- If there is no fluid restriction, increase your intake (8-10 glasses of water per day) and encourage sips of fluid throughout the day.

- Evaluate and record bowel movements.

**5. Stress Incontinence:**

- Assess adequate hygiene practices, need for skin care, and deterioration of skin integrity.

- Teach urogenital area hygiene practices and skin care.

- Teach techniques that strengthen the sphincter and structural supports of the bladder.

- Limit your intake of bladder irritants such as caffeine and soda.

- Inform him not to reduce his fluid intake (at least 1.5 liters).

- Ask the elderly person and their family to report signs and symptoms of urinary tract infection.

- Identify and eliminate the reasons that prevent the elderly from reaching the toilet.

- Inform the elderly and their families about the side effects of drugs.

- Organize bladder training / toileting schedules.

**6. Risk of Injury:**

- Monitor the level of consciousness and neurological status during hospitalization

- Ask them to complete a three-step task (e.g. “put your right hand on your chest, shake it with your left hand and then raise your eyebrows”)

- Use the confusion assessment method to determine the presence or absence of delirium/confusion

- Identify the cause of acute confusion

- Evaluate your vital signs

- Monitor pain with a scale, if it is not possible to assess with a pain scale, evaluate non-verbal cues such as frowns, grimaces, clenched fists

- Apply pain treatment and monitor your behavior according to the physician's request

- Track fluid intake and output every eight hours

- Evaluate laboratory findings

- Review all medications she uses, including over-the-counter drugs

- Put routinely used items in an easily accessible place for the elderly

- If he wears glasses and hearing aids, have them wear them, keep them close to the bedside and within easy reach

- Ensure that an item that is important to the elder is brought

- Check the patient at least every 30 minutes and each time they pass from their room, if possible, place the elderly person in a room close to the nurse's desk, and provide a safe and non-stimulating environment

- Ensure that a clock written in large numbers and a large calendar are placed in the room, verbally remind the patient of the date and day when necessary

- Allow him to listen to music and watch TV

- Explain the attempts made and the situation using simple language

- Check at least every 30 minutes if he has a persistent or serious cognitive problem

- You can store your tubes, serum sets, etc. hide them so they don't see

- Apply medications according to the physician's order

- If necessary, do the physical restraint of the elderly carefully and in accordance with the hospital policy.

**7. Risk of Infection:**

- Monitor signs and symptoms of infection (body temperature, pulse rate, respiratory sounds, rales, secretion, appearance of urine, skin temperature, etc.)

- Evaluate the factors that increase susceptibility to infection (advanced age, suppression of the immune system, malnutrition, etc.)

- Monitor laboratory findings (CBC, granulocyte count, blood and urine culture, serum albumin and protein values, etc.)

- Assess the skin for tearing, scraping, redness or ulceration

- Evaluate the color and quality of your administration

- Avoid the use of urinary catheters as much as possible

- Observe individual hygiene practices to protect against infection.

- Educate the elderly and their families about the disease and why treatment increases the risk of infection.

- Educate the elderly and their families on hygiene/isolation precautions-practices to prevent infection

- Give information about what needs to be done to strengthen the immune system function (adequate and balanced diet, adequate hydration, rest, regular physical activity, etc.)

- Explain the reasons, benefits and side effects of vaccination (influenza and pneumonia) especially for the elderly and their families.

- Suggest reducing contact with other people during the influenza epidemic

- Teach proper hand washing techniques

- Tell visitors to wash their hands when entering and leaving the patient's room

- Avoid cross-contamination by not assigning the same nurse to care for another infected patient and by not putting the patient in the same room with an infected patient

- Apply appropriate isolation measures (respiratory isolation, etc.) for the elderly.

- Maintain "universal precautions / CDC" to prevent infection

- Since signs and symptoms of infection can be hidden in old age, other than typical signs and symptoms of infection, confusion etc. watch for symptoms

**8. Impaired Physical Mobility:**

- The level of mobilization in bed and standing and the level of ability to perform daily living activities will be evaluated.

- His emotional, social and spiritual reactions to activities will be evaluated.

- His motivation and willingness to increase his activity will be evaluated.

- Causes of fatigue will be determined together with the elderly

- Nutritional status will be evaluated

- Activity tolerance will be evaluated

- If the patient has had surgery, assess understanding of the post-operative activity and exercise program

- Evaluate his knowledge of movement and, if using an assistive device, his knowledge of moving with the relevant vehicle

- Teach him/her to take proper position/movement

- Information about ROM exercises will be given

- If he/she uses/will use an assistive tool, he/she will be taught to use it correctly.

- It will be possible to use a trapezoidal bar on the bed

- Assist the elder during standing up or transferring until he/she can move safely

- Encourage her to ask for the help she needs

- He will be taught how to do activities of daily living by supporting his independence.

- Effective pain management will be provided

- The cardio-respiratory response to the activity (tachycardia, dyspnea, sweating, pallor/pallor, etc.) will be monitored.

- Sleep pattern will be monitored and sleep time will be recorded

- Measures to conserve energy, such as keeping frequently used objects within easy reach, will be taught.

- Extra time will be allowed for treatments and activities of daily living

- Help him to change positions, sit up, hang his feet off the bed, stand up and move as slowly as he can tolerate

- Helping to perform daily living activities

- It will be ensured that the activities are performed partially, not all at once.

- A comprehensive physical assessment will be made

- Chronic disease status will be evaluated

- Laboratory findings will be evaluated

- He will be allowed to feel fear, anger, hopelessness, frustration, hospitalization and health concerns

- Age-related changes, illness, drug use, etc. others will be announced

- Collaborating with speech therapist, dietitian, physical and occupational therapist, social services when necessary

**9. Risk of Ineffective Tissue Perfusion:**

- Signs and symptoms related to decreased cardiac output will be monitored.

- The color and temperature of the skin (if the skin is pale and mottled, there may be arterial obstruction if there is no pulse) will be evaluated

- Extremities will be evaluated for pain

- Capillary refill will be evaluated

- Foot and leg ulcers and gangrene will be checked.

- ECG changes will be monitored

- Hemoglobin, hematocrit and platelet counts will be monitored

- Consciousness will be monitored

- Tissue hypoxia (mixed venous oxygen saturation) signs and symptoms will be monitored and evaluated

- Oxygen support will be provided according to the physician's request

- Adequate organ perfusion will be provided with medication and fluid therapy according to the physician's request.

- Fluid intake and output will be tracked

- If the deterioration in tissue perfusion is caused by anemia, blood transfusion will be performed according to the physician's request.

- Bilateral dorsalis pedis, posterior tibialis and popliteal pulses will be taken

- To be monitored by monitoring if necessary

- Pharmacological and non-pharmacological interventions will be applied to provide pain control

- Nursing interventions to ensure skin integrity will be applied,

- Provide adequate hydration

- Appropriate position will be given for adequate tissue perfusion

**10. Hypothermia:**

- Monitor body temperature with an oral or tympanic thermometer.

- Do not use the axillary thermometer, as the decrease in peripheral circulation and subcutaneous fat in the axillary area due to aging may cause an incorrect body temperature reading.

- Evaluate and record your mental state.

- Be careful with the use of sedatives, muscle relaxants and hypnotic (including anesthetics) drugs as they can reduce tremor.

- Make sure that there are no exposed parts of the elderly during diagnostic procedures and cover the elderly with a blanket while being sent to the relevant unit for diagnosis.

- Warm up slowly if slightly hypothermic.

- If the elderly person's temperature drops below 35 °C, give them warm oral or IV fluids to keep them warm.

- Watch for signs of rapid rise in body temperature.

- If there is no increase in body temperature despite the attempts made in hypothermia, possible sepsis, hypoglycemia, hypothyroidism, etc. Collaborate with the physician.

- Apply the drugs according to the physician's order, evaluate the effects, side and opposite effects of the drugs.

**11. Disruption in Sleep Pattern:**

- Assess individual, environmental and therapeutic factors related to sleep.

- Evaluate, monitor and record your nighttime sleep routine.

- Monitor your activity level.

- Provide wakefulness activities/support when necessary to limit the elderly's sleeping during the day.

- Provide the transition to sleep with relaxing applications such as massage, positioning and touching.

- Regulate environmental stimuli to maintain the normal day-night cycle.

- Measure vital signs, administer medications and organize activities such as toileting.

- Maintain a calm and quiet environment and reduce interruptions in sleeping hours.

- If there is pain, apply analgesics according to the physician's order.

- Leaving the bed if unable to sleep within the first 30 minutes, engaging in non-strenuous activities such as listening to music and reading a book, and not being exposed to bright light.

- Paying attention to exposure to enough light during the day

- If there is excess weight, it should be given.

- Not sleeping more than 50 minutes during the day.

- Avoiding drugs not prescribed by the physician

- Evaluate the symptoms such as anxiety, perception disorder, slowdown in reactions and irritability, etc., which occur in the elderly due to sleep deprivation.

**12. Aspiration Risk:**

- Assess the swallowing reflex by placing your thumb and forefinger on either side of the laryngeal prominence and telling the elderly to swallow

- Evaluate the burping reflex by gently touching the arch of the palate to one side and then the other side and record these findings

- Record food consumption (amount and density), where you put the food in your mouth, how you chew before swallowing, and the time elapsed before swallowing

- Assess the swallowing reflex by placing your thumb and forefinger on either side of the laryngeal prominence and telling the elderly to swallow

- Evaluate the burping reflex by gently touching the palatal arch to one side and then the other side with an abslant and record these findings

- Record food consumption (amount and density), where you put the food in your mouth, how you chew before swallowing, and the time elapsed before swallowing

- Watch the elder while swallowing

- Monitor the elderly for choking or coughing before, during or after swallowing

- Evaluate for abnormal breathing sounds (rales, rhonchus, wheezing, etc.), dyspnea, cyanosis, hyperthermia, and worsening of consciousness

- Pay attention to whether you are holding food on the corners of the mouth

- Assess the intensity of intraoral secretion

- If swallowing reflux is impaired, raise the head 45° during swallowing, in the elderly with hemiplegia, raise the head to the unaffected side

- Let him rest before meals

- Make sure the denture is properly placed

- Tell the demented old man to chew and swallow every bite

- Give him enough time to finish eating and drinking

- Allow someone to stay with the elderly while eating or drinking

- Encourage him to do breathing and coughing exercises every 2 hours while awake and every 4 hours during the night

- Have your blowjob ready

**13. Risk of Fluid Volume Deficiency:**

- Evaluate and record the amount, color and frequency of any discharge of fluid, including urine, diarrhea, vomit or other drainage

- Evaluate and save skin turgor

- Monitor fluid intake, encourage 2-3 liters of fluid intake per day if there are no contraindications; specify fluid intake targets for day, evening, and night shifts

- Assess level of consciousness, including orientation, ability to follow commands, and behavior

- Weigh the elderly at the same time of day (usually before breakfast) using the same measurements and clothes

- Assess self-hydration and drinking ability, keep liquids within easy reach, use lidded cups to reduce the worry of spills

- Monitor for signs and symptoms of dehydration or hypervolemia

- Identify factors that increase the risk of fluid volume imbalance

- Track vital signs

- Monitor lab findings

- Accurately record the amount of fluid taken in and out

- Keep track of your daily weight

- Perform oral care at frequent intervals

- Ensure that the elder has easy access to the toilet, urinal, or sink at least every two (2) hours while awake and every four (4) hours at night

- Answer the call system immediately

1. **MESENTERİC ISCHEMİA**

**Learning objectives:**

**At the end of this session the participants are expected to:**

1. Follow vitals at regular intervals, and be aware that normotensive values may not be normal values for elderly patients.

2. The threshold for examination for abdominal pain should be low in elderly patients

3. Know that fatal diagnoses can occur under symptoms such as flank pain, diarrhea, stomach pain.

4. Consider to see ECG in elderly patients even with symptoms other than cardiac causes

5. Consider AMI with normal lactate and D-Dimer tests taken in the acute period (acute mesenteric ischemia).

6. Know that it is not necessary to rush to discharge the patient who is not relieved and be conscious of the re-evaluation of the patient

**CASE INFORMATION**

Time: 14:30

You work as a doctor in the yellow area. A new patient came; you went to see him.

A 76-year-old female patient.

Main complaint: vomiting, diarrhea

Vitals: BP: 100/75 mm Hg, Pulse: 115/min, O_2_ saturation: 98%, Fever: 36.6 C^o^

The patient has pain, showing his tummy.

Story: She ate lunch. Chicken, rice. About 40 minutes later, abdominal pain started suddenly. After 10 minutes, she could hardly reach the toilet and diarrhea started. Lots of water, no blood. She felt dizzy when she got up from the toilet, but it passed quickly. Relatives brought them to the ED within 15 minutes. She vomited once on the way.

• I have a lot of pain, can you take painkillers? says.

Background: Diabetes exists, but it is controlled.

Drugs: Glyfor

Physical examination: GCS: 15; place-time orientation is complete.

There is no pathology in lung sounds.

Abdomen is comfortable in abdominal examination; widespread tenderness but no defense or rebound. The abdomen is not distended. There is no palpable mass in the west. Peripheral pulses are on.

You want her tests because she is an old patient. You order analgesics.

Analgesic was given. Blood gas results; venous: Ph:7.35, Po2: 45, CO2:30, Hco3:22, lactate: 2.1

40 minutes passed. The pain has lessened slightly. She wants painkillers.

Another analgesic was administered. Blood tests are as follows:

Hb: 10.1 g/dl

Plt: 132000

Leukocytes: 12500

Na/K/Cl: 136/3.5/108

ALT/AST: 28/32

BUN/Kr: 20/1.0

Lipase: 80

TIT: 5 leukocytes, 1 erythrocyte, nitrate (-), Esterase (-)

Stool: watery consistency, 5 leukocytes, 10 erythrocytes

As a result of the examinations, you thought about enteritis, wrote a prescription and went to the patient. Her relatives went to the canteen. You also told the patient that there was nothing important, that you had prescribed her medications, that she should use them, and that she could be discharged. You told the nurses that the patient would be discharged when her relatives came, that the intravenous line should be removed and you left to take care of other patients. While the patient's vascular access was taken, the patient said that she had abdominal pain and asked for painkillers. The nurse on duty said that enough painkillers were given, that more of it would harm her, that there may be pain in diarrhea cases, that her doctor discharged her, that she should use the drugs. The patient told her relatives that she wanted to go to the toilet again. They took their patients in wheelchairs and took them to the toilet.

3 minutes later, shouts were heard from the toilet. The patient passed out. The staff put the patient in a wheelchair and brought her to the red area. Here, the vitals of the patient, who was transferred to the stretcher, were examined. Vascular access was requested, but no full vein was found. Opened on 3rd try.

Vitals taken: BP: 70/43 mm Hg, Pulse: 145/min, O_2_ saturation: 97%, Fever: 36.7 C^o^.

Fingertip blood sugar: 190 mg/dl

ECG: 140/min AF+

If blood gas is requested: Blood gas, venous: pH:7.32, PO_2_: 35, CO_2_: 25, HCO_3_:20, lactate: 5.0

Fluid resuscitation was started quickly.

When the patient's relatives went to give information, the patient's relative said that he saw bloody stool in the toilet. IV opaque CT was performed in the patient who presented with arrhythmia and symptoms of enteritis. It was reported as emboli material+ at the 1st cm of the superior mesenteric artery. The patient, who was consulted with general surgery, was taken to emergency operation.

Tasks:

1. When the approaches and behaviors of the doctors, nurses and staff are evaluated, where do you think there is a lack of or wrong behavior? Please specify separately under the heading of doctor, nurse, hospital staff and 112 staff.

2. How should abdominal pain be approached in the elderly patient? Why should the approach be different from the adult?

3. Should an EKG be taken only for those with chest pain? Please state briefly.

4. Does the normal laboratory values indicate that everything is normal? Please state briefly.

1. **a) Doctor's process:**

- Even if the vitals are within the normal range, the measured value may not be normal for the elderly patient, depending on the drug used, etc. tachycardia response may not develop. You need to be vigilant about this. The shock index (heart rate/systolic) for this patient was 115/100 equal to 1.15 (normal 0.7-0.9), high. This patient should be evaluated for shock. Vital follow-up should be done, vitals should be taken before discharge. In this patient, only the arrival vitals were taken, and after the examination, the patient was tried to be discharged without even taking the last vitals.
- Myocardial infarction is among the prediagnoses of abdominal pain. It is frequently written in geriatric emergency books, especially for the elderly patient, that "even if the problem does not appear cardiac, the underlying cause is most likely cardiac". ECG acquisition threshold should be kept low in elderly patients. If the ECG had been taken on arrival in this patient, atrial fibrillation would have been seen and the diagnosis would have been easier.
- Dizziness when getting up from the toilet should be a warning in terms of orthostatic hypotension. Patient probably had a hypotensive episode. According to the patient's history, conditions that cause hypotension such as septic shock, cardiogenic shock, hypotensive attack caused by drugs, hemorrhagic shock (gastrointestinal bleeding), and obstructive shock should be reviewed. In the aforementioned case, everything was connected to the diagnosis of enteritis and the diagnosis was skipped without making additional preliminary diagnoses.
- Only analgesic was ordered to the patient. Fluid was not started in the patient who described severe enteritis and orthostatic blood pressure with a high shock index. The doctor did not even feel the need to learn the patient's control vitals. (Pain can also cause tachycardia, but pain and vital monitoring should have been done in this patient.)
- Severe abdominal pain inconsistent with physical examination is typically a finding that needs attention in terms of mesenteric ischemia. The patient repeatedly requests analgesics and there is no relief in terms of pain. We need to re-examine our diagnoses in patients whose pain does not go away. It means that we are likely to miss an important diagnosis. Do not discharge the patient who is not relieved; re-examine, re-examine. Perform the necessary examinations and imaging again.
- Time is required for the development of lactate and metabolic acidosis in blood gas. If the result of the initial tests is normal, request again if clinical replication is required.
- Talk to the patient and give information, but the patient may have an undiagnosed dementia or he/she may not have understood but said he/she understood so as not to be embarrassed. Explain again when relatives come. If you have time to explain at once, tell them when their relatives arrive. Many researches have been done on this subject. When only the patient is informed about the use of treatment, outpatient follow-up, and discharge recommendations such as circumstances where emergency service referral is required are not well understood, the treatment processes of the patients are disrupted, there are more frequent emergency service applications and the need for intensive care may arise. Make sure that you agree on a common denominator with the relatives of the patients. In addition, after verbally explaining the discharge recommendations, it is recommended to give written information to the patients and their relatives.

**b) Nursing process:**

**Falling risk:**

- Assess the risk of falling

- Evaluate orthostatic hypotension

- Put on a fall risk marker

- Use stretcher borders and locks

- Review the hospital's "patient transfer" protocol.

- Keep the hospital bed at the lowest level at all times

- Ensure that family and relatives stay with the elderly

- Accompany the elderly during his mobilization if necessary

**Fluid volume deficiency:**

- Watch for signs of dehydration.

- Monitor vital signs.

- Accurately record the amount of fluid taken in and out

- Answer the call system immediately

- Monitor lab findings

**Acute pain:**

- Reduce ignorance: explain to the person the cause of the pain, if known

- Teach non-invasive methods of reducing pain:

- Relaxation, stretching, hot/cold application

- Administer prescribed analgesics

- Evaluate efficacy 30 minutes after analgesic administration.

**Failures:**

I. Vital follow-ups were not taken in our patient.

ii. The patient's pain was not relieved. The patient is not relieved. The doctor was not informed again.

iii. Discharge vitals were not taken. If he had been found to be hypotensive, vascular access would not have been drawn. For this reason, there was a problem in reopening the vascular access of the patient who was taken to the red area.

**c) Process of the personnel:**

A wheelchair is not suitable for the transport of the unconscious patient. The patient may fall. It should be taken to the stretcher and the side barriers of the stretcher should be raised. The decision of the staff to draw the unconscious patient to the red area, not the yellow area, is a good decision. This patient will require urgent intervention, including intubation.

**d) 112 personnel:**

“If the patient was taken from her home, it will be beneficial for the patient to make a habit of taking a look at the home environment, how his relatives behave, and how the house is cleaned. Clues of a possible neglect/abuse situation can be caught by looking at the home environment. It should be asked whether the patient has a physical disability, and if the patient is using it, the patient's relatives should be told about bringing the hearing aid, glasses and walking stick with the patient. If any, previous epicrisis, medication bag or list should be taken with the patient.

**2.** Abdominal pain is a difficult process to diagnose in the elderly patient. Confronting the patient's co-morbid conditions, medications, inability to express himself well, and situations that mask the physical examination, such as dementia/delirium/change of consciousness, may conceal the occurrence of classical findings. Although flank pain makes the diagnosis of renal colic in the foreground in the young patient, it brings the diagnoses such as aortic dissection, aortic aneurysm rupture, renal infarction to the fore in the elderly patient. Under the sudden onset of abdominal pain, mesenteric artery embolism should be considered in addition to the previous diagnoses. Mesenteric ischemia may initially present with signs of enteritis. Since the history and physical examination are often not sufficient, the physician should keep the examination and imaging request threshold low. In a patient whose pain does not go away, an important diagnosis is probably missed. The patient should be questioned again, necessary examinations and imaging should be requested.

**3.** While there may be primarily cardiac causes underlying the complaints of elderly patients, secondary consequences of cardiac causes may also cause the patient's complaints. Do not wait for the typical chest pain to take an EKG in the elderly patient. Keep your EKG acquisition threshold low. Arrhythmia, ischemia findings, QTc time should be checked. Otherwise, you may be missing an important diagnosis.

**4.** Just like vitals, normal laboratory findings do not indicate that everything is going well. It may take time for some values to change significantly. A patient with normal lab values may develop cardiac arrest within minutes. Lab values guide us, but they should be evaluated together with anamnesis and physical examination, not alone.
