# Supplementary material for: Training interprofessional teams in geriatric emergency medicine: A modified team-based learning approach
Source: Heliyon. 2024 Feb 7;10(4):e25099. doi: 10.1016/j.heliyon.2024.e25099 (PMC10877185; doi:10.1016/j.heliyon.2024.e25099)
Supplement: Multimedia component 3 [file mmc3.docx]

**Appendix 3. Feedback forms**

**Trainer feedback form**

| **PHYSICAL CONDITIONS** |
| --- |
| 1. Physical conditions in the learning environment were suitable. |
| **PRELIMINARY PREPARATION AND READINESS OF PARTICIPANTS** |
| 2. Time allocated for my preparation was sufficient |
| 3. The reading material provided for the participants adequately covered the main concepts |
| 4. Readiness assurance test questions were directly related to learning objectives |
| 5. Time allocated for readiness assurance tests and discussions was sufficient |
| **TEAM DYNAMICS AND DISCUSSIONS** |
| 6. Team members communicated with each other well and encouraged each other to express their ideas |
| 7. Team members actively discussed multiple perspectives before deciding on a final answer. |
| 8. Each team member made an effort to participate in the discussion. |
| 9. Compared to lecture at the beginning of the day, participants were more engaged with team discussions |
| 10. Team members criticized and were criticized without making it personal |
| 11. Team members showed respect to different viewpoints |
| 12. Participants maintained their interest during team discussions |
| **GENERAL** |
| 13. Training process covered all of the predetermined topics. |
| 14. I found this training program more productive than standard didactic courses |
| 15. I found this training program more enjoyable than standard didactic courses |
| 16. I prefer teaching all topics in this program’s format. |
| 17. I am satisfied with the implementation of the training program in this form |
| **Additional comments:** |

**Participant feedback form**

| **ORGANIZATION, INFRASTRUCTURE AND FACILITIES** |
| --- |
| 1. The information given about the instruction method before the training was sufficient for me to understand the flow of the program correctly. |
| 1. The training program was well organized (time allotted, timing of breaks, conducting readiness tests and discussions, etc.). |
| 1. Physical conditions in the learning environment were suitable |
| **PRELIMINARY PREPARATION AND READINESS** |
| 1. Self-study materials provided and suggested were sufficient for me to acquire the necessary preliminary knowledge. |
| 1. Individual/team test content was challenging enough to start discussion. |
| **DISCUSSIONS** |
| 1. Team assignments (case scenarios) facilitated my learning process positively |
| 1. The discussion of all possible solutions facilitated comprehensive learning |
| 1. This training program helped us show a more systematic and logical approach to the elderly patients under emergency conditions. |
| **TRAINERS** |
| 1. The trainers helped us to better comprehend the subject by providing feedback, discussion, and explanations. |
| 1. The trainers contributed to our learning in the discussion sessions as well as the lecture. |
| 1. The trainers successfully managed the entire training process. |
| **GENERAL** |
| 1. This training program increased my interest in the subject. |
| 1. I was able to focus on the topics covered in the discussions for a longer period of time compared to the lecture, where I only participated as a listener. |
| 1. I was able to focus on the subject covered in discussions for a longer period of time compared to the lectures |
| 1. I was engaged with the learning process more actively in this training program compared to other programs in which I participated only as a passive listener. |
| 1. I think that the knowledge I gained through this training program will be more permanent. |
| 1. Overall, I am satisfied with the training program I attended today. |
| **Additional comments:** |
